# Fabrication of Lanthanum Strontium Manganite Ceramics via Agar Gel Casting and Solid State Sintering

**DOI:** 10.3390/ma12060848

**Published:** 2019-03-13

**Authors:** Shiyu Zhang, Cheng Peng, Chengzhi Guan, Guoping Xiao, Jianqiang Wang

**Affiliations:** 1 Department of Molten Salt Chemistry and Engineering, and Key Laboratory of Interfacial Physics and Technology, Shanghai Institute of Applied Physics, Chinese Academy of Sciences, Shanghai 201800, China; zhangshiyu@sinap.ac.cn (S.Z.); guanchengzhi@sinap.ac.cn (C.G.); xiaoguoping@sinap.ac.cn (G.X.); 2 University of Chinese Academy of Sciences, No.19(A) Yuquan Road, Shijingshan District, Beijing 100049, China

**Keywords:** gel casting, lanthanum strontium manganite, agar, ceramic formation, solid state sintering, cathode-supported SOFCs

## Abstract

Fabricating lanthanum strontium manganite (LSM) ceramics with certain shapes is important for the design and construction of high-temperature energy conversion and storage devices. Here, we describe a low-cost and environmentally friendly method for fabricating LSM ceramics via agar gel casting and high temperature sintering. This new approach uses temperature tuning to fabricate LSM gel bodies, not only by manufacturing in the secondary process but also by remolding and recycling during the gel casting process. The effect of the sintering temperature on the properties of LSM ceramics was investigated as well. As a result, the porosity and compressive strength of LSM ceramics sintered at 1000 °C are ~60% and 5.6 MPa, respectively. When the sintering temperature increases to 1200 °C, the porosity decreases to ~28%, whereas the compressive strength increases to 25 MPa, which is able to meet the requirement of cathode-supported SOFCs (solid oxide fuel cells).

## 1. Introduction

As a representative of perovskite-type oxide system ceramics, lanthanum strontium manganite (La_1−x_Sr_x_MnO_3_, LSM) attracted intensive interest because of their outstanding electrical conductivity and catalytic activity [1,2,3,4,5], that make them promising cathode materials for high-temperature energy conversion and storage devices, such as solid oxide fuel cells (SOFC) [6,7,8,9] and molten salt batteries [10,11,12]. In particular, forming LSM ceramics is crucial in the design and fabrication of cathode-supported SOFCs [13], which is associated not only with structures and shapes but also with manufacturing costs. Currently, a variety of forming techniques, including dry pressing [14,15], dip coating [7], screen-printing [16], tape casting [17], gel casting [18,19,20] and extrusion molding [21], have been utilized to fabricate porous LSM ceramic electrodes for planar, tubular and honeycomb cathode-supported SOFCs. However, it is still a challenging task for the development of advanced shaping technologies to meet urgent demands of geometrical optimization and cost reduction in the rapidly growing area of cathode-supported SOFCs in micro/meso-scale.

Gel casting is a near-net shape ceramic forming technique that has been widely employed to fabricate electrode-supported substrates for SOFCs [22,23]. Recently, porous LSM cathode tubes in centimeter scale have been fabricated via a typical gel casting process with the binder of polyacrylamide. In order to gelation, many chemicals, i.e., acrylamide, N,N’-methylenebisacrylamide (MBAA), N,N,N’,N’-tetramethylethylenediamine (TMED) and ammonium persulfate (APS) were used as monomer, crosslinking agent, catalyst and initiator, respectively, and 24 h is essential for fully polymerization [24]. Agar gel casting provides an alternative approach that has been utilized to fabricate anode-supported substrates for SOFCs. As a kind of natural polysaccharide, agar is a non-toxic gelling agent for aqueous ceramic suspensions, which is able to gelation activated by simple cooling below the glass transition temperature instead of using catalyst, initiator and crosslinking agents as in the case of synthetic monomers. Furthermore, agar offers high gel strength at low concentration, and thus the gel body is capable of additive manufacturing in the secondary process for fabricating ceramic bodies with certain shapes [25]. In the present work, porous LSM ceramic bodies were fabricated using agar gel casting and solid state sintering. The effects of gel casting and thermal annealing conditions on the microstructures, dimensions, porosities and mechanics were investigated. Preliminary secondary processing capability trials, i.e., cutting and drilling in sub-millimeter scale were performed, and the successful green machining of gel casting billets was also demonstrated.

## 2. Materials and Methods

The raw material LSM ceramics with the chemical structure of La_0.65_Sr_0.35_MnO_3_, were purchased from Ningbo SOFCMAN Energy Technology Co., Ltd (Ningbo, China). The LSM nanopowders with the 200–300 nm diameter for gel casting were obtained via ball milling treatment with 4:1 ball-to-powder weight ratio. Agar, polyethylene glycol (PEG-600) and polyacrylic acid (PAA) of 50% aqueous solution were purchased from Aladdin reagent Co., Ltd (Shanghai, China). The ball mill instrument is BMT-30D benchtop ball mill, which was purchased from Hsiangtai Machinery Industry Co., Ltd (New Taipei City, Taiwan). The agar aqueous solution with concentrations of 1 wt%, 2 wt%, 3 wt%, 4 wt%, 5 wt%, respectively, which was prepared for suspending LSM powders at a constant temperature of 98 °C, and the solid content was controlled at 60 wt% for well-dispersion and moderate-viscosity. PAA and PEG were used as a dispersant and plasticizer with the mass ratio of 2% and 1.5% to LSM powders, respectively, for improving dispersibility and plasticity. The as-prepared LSM slurry was filled into glass tubes and then cooled down to room temperature to form gel bodies. After removing the LSM gel from the glass tube, the gel bodies were dried at 50 °C for 2 days, and then the obtained dehydrated bodies were pyrolyzed and sintered in the furnace (HTF 1800, Carbolite, Sheffield, UK) with a temperature rising rate of 1 °C per min. In a typical pyrolysis process, the dehydrated bodies were treated at 200 °C for 2 h to remove water, and then treated at 550 °C for 2 h to decompose organics. In the sintering treatment, the LSM bodies were sintered for 6 h at 800, 900, 1000, 1100, 1200 °C respectively.

X-ray diffraction spectra (XRD) was carried out with an X-ray diffractometer (Bruker AXS, D8 Advanced, Billerica, MA, USA) using Cu Kα radiation (λ = 1.5418 Å) generated at 40 kV and 40 mA over a 2θ range of 20° to 90° with a sampling width of 0.02° and a sampling speed of 10° per min. The scanning electron microscopy (SEM) images of LSM ceramics at different sintering temperatures were collected by LEO 1530VP, ZEISS (Oberkochen, Germany), and the high-resolution transmission electron microscopy (HR-TEM) images and selected area electron diffraction (SAED) results were obtained by Tecnai G2 F20 S-TWIN, FEI (Hillsboro, OR, USA). The thermogravimetric analysis of the dehydrated body was tested by TG, TG209F3, NETZSCH (Selb, Germany), with the heating rate of 10 °C per min from room temperature to 900 °C. Both of the volume shrinkage and relative density were measured by a fast draining method. Compressive strength data were obtained using Zwick Roell materials testing machine (Z250, Zwick, Ulm, Germany), with the compression speed of 2 mm/min. The pore size distribution and porosity were investigated by mercury injection apparatus (AutoPore IV 9500 V1.09, Micromeritics Instrument Corp., Norcross, GA, USA), with a pressure range from 0.1 to 60,000 psia. The specific surface area was measured by the BET method (BET, Quantachrome Instruments, Boynton Beach, FL, USA), with an analysis gas of N_2_ and bath temperature of 77.3 K.

## 3. Results

### 3.1. Fabrication of LSM Sintered Bodies

**Figure 1:**
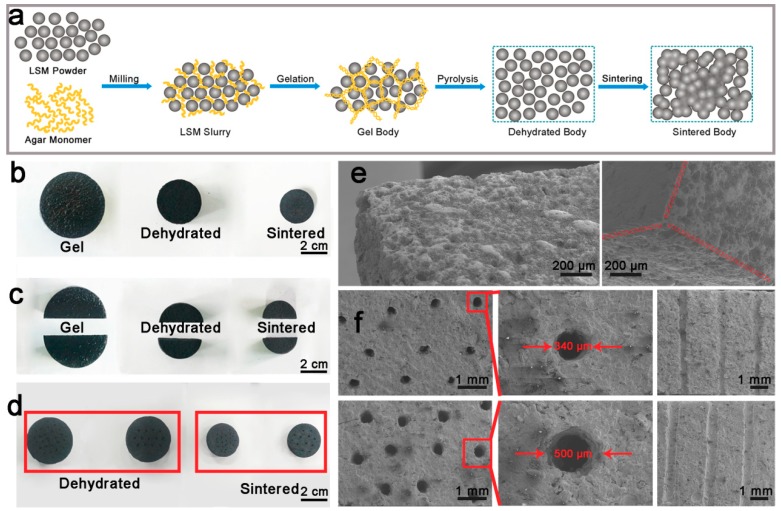
(**a**) Schematic drawing of the fabrication of lanthanum strontium manganite (LSM) ceramics via agar gel casting and high temperature sintering; (**b**) the top view of gel, dehydrated and sintered LSM cylinders, respectively; (**c**) the top view of the cut gel, dehydrated and sintered body, respectively; (**d**) the top view of the drilled gel bodies with different hole-diameters after dehydration and high-temperature sintering treatment, respectively; (**e**) SEM images of the cut gel body after high-temperature sintering treatment; (**f**) the SEM images of the drilled gel bodies with different hole-diameters after high-temperature sintering treatment, the magnified holes and the cross sections, respectively.

LSM ceramic bodies were fabricated via gel casting and solid state sintering, as shown in Figure 1a. In detail, LSM slurry was prepared by suspending La_0.65_Sr_0.35_MnO_3_ nanopowders in agar solutions at 98 °C, with the use of polyacrylic acid (PAA) and polyethylene glycol (PEG) as a dispersant and plasticizer, respectively. Then, we cast the as-prepared LSM slurry into molds (e.g., glass tubes) for gelation with a treatment of cooling below 4 °C. After removing the LSM gel from the mold, the as-fabricated cylindrical gel bodies dehydrated at 50 °C and subsequently sintered under high temperatures to form porous LSM ceramic bodies. Figure 1b shows the typical shapes of LSM gel, dehydrated and sintered body, respectively. Here, we observed that the cylindrical shape of LSM gel body was well-retained after both dehydration and high-temperature sintering treatment. Furthermore, neither the dehydrated nor sintered bodies have a fracture or crack, even though the diameter of LSM gel body decreases from ~3.5 to ~2.5 cm after dehydration and further to ~1.8 cm after sintering treatment at 1200 °C. This firmly indicates that agar gel casting is suitable for fabricating LSM ceramic bodies.

In order to explore the ability of manufacturing in the secondary process, we cut the cylindrical LSM gel body into two parts and drilled hole-arrays with different diameters on LSM gel bodies, respectively. Similar to the pristine cylindrical LSM bodies, the shapes of both cut and drilled LSM bodies in Figure 1c,d have been well-retained after dehydration and high-temperature sintering treatment in spite of the significant volume shrinkage. Scanning electron microscopy (SEM) images clearly demonstrate the sharp straight edge in Figure 1e and hole-arrays in Figure 1f of the cut and drilled LSM bodies after the sintering treatment at 1200 °C, respectively. Importantly, no fracture or crack occurs on the surface of sintered bodies or at the cut edge and hole edge, even inside the holes. This strongly implies the remarkable capability of agar-based LSM gel bodies for manufacturing in the secondary process. Of note, the hole-diameter ranges from 340 to 500 μm, appropriate for fabricating cathode-supported SOFCs in sub-millimeter scale.

Moreover, thermogravimetric analysis (TGA) of the dehydrated body was conducted to investigate the removal of agar during the heating stage. The TGA curve in a range from room temperature (RT, ~25 °C) to 900 °C depicts that the degradation of the dehydrated body can be roughly divided into three weight-loss stages of RT~280 °C, 280~460 °C and 460~820 °C, respectively, as shown in Figure S1 (Supplementary Materials). In the first stage, the weight of the gel body decreases ~2.7% as the temperature rises to 280 °C, corresponding to water evaporation. Furthermore, we observed a weight loss of ~3.4% in the second stage at 280~460 °C and a weight loss of ~3.5% in the third stage at 460~820 °C, which should be attributed to the pyrolysis of organic materials including PEG, PAA and agar. Importantly, the weight remains nearly invariable when the temperature is higher than 800 °C, strongly indicating that all the organic materials have been completely decomposed before the high-temperature sintering treatment of LSM powders.

### 3.2. Microstructures of LSM Sintered Bodies 

**Figure 2:**
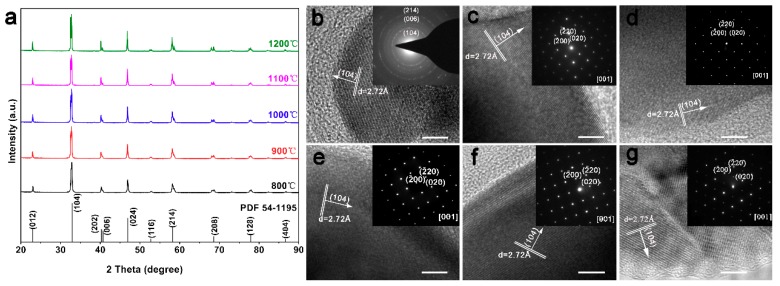
(**a**) The XRD spectra of LSM sintered bodies at different sintering temperatures; (**b**) the high-resolution transmission electron microscopy (HR-TEM) and selected area electron diffraction (SAED) image of pristine LSM powders; (**c**–**g**) The HR-TEM and SAED images of LSM sintered bodies at different sintering temperatures, 800 °C (**c**), 900 °C (**d**), 1000 °C (**e**), 1100 °C (**f**), 1200 °C (**g**), respectively. The scale bars in the TEM images indicate 5 nm.

Next, we studied LSM sintered bodies via X-ray diffraction (XRD). Figure 2a presents the XRD patterns of LSM sintered bodies at different sintering temperatures, exhibiting peaks at 2θ values of 22.9°, 32.7°, 40.2°, 40.5°, 46.8°, 52.8°, 58.2°, 68.6°, 77.9° and 86.7°, which can be attributed to the crystal planes of (012), (104), (202), (006), (024), (116), (214), (208), (128), and (404) in the ABO_3_ perovskite structure of lanthanum strontium manganite (La_0.65_Sr_0.35_MnO_3_), respectively, according to the reference pattern JCPDS card 54-1195. Furthermore, the relative intensities of diffraction peaks in XRD patterns remarkably increase by raising the sintering temperature, implying that the high sintering temperature improves the crystallization of LSM sintered bodies.

High-resolution transmission electron microscopy (HR-TEM) with selected area electron diffraction (SAED) was used to further analyze the crystal structure of the LSM sintered bodies. The lattice planes with a distance of ~2.72 Å can be observed in the HR-TEM images of pristine LSM powders in Figure 2b and LSM sintered bodies at different sintering temperatures in Figure 2c–g inset, which is in good agreement with the (104) plane of the perovskite La_0.65_Sr_0.35_MnO_3_ structure. Moreover, the SAED patterns in Figure 2c–g inset, measured from [1] zone-axis demonstrate that the lattice parameters are a = 5.4 Å, b = 5.4 Å and c = 15.3 Å, which are consistent with data in other reports.

**Figure 3:**
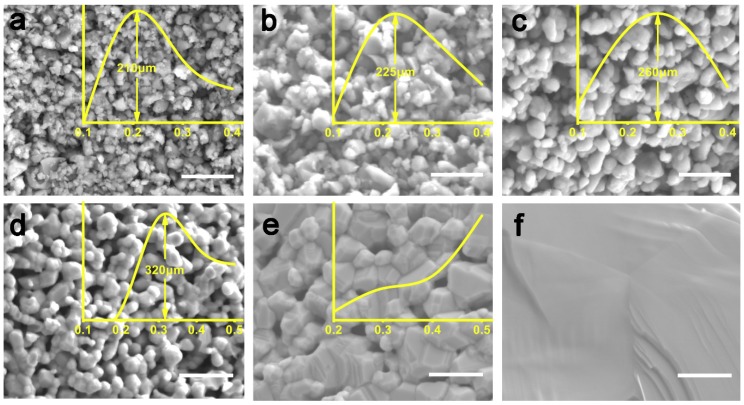
SEM images of LSM ceramics of unsintered (**a**), and sintered at 800 °C (**b**), 900 °C (**c**), 1000 °C (**d**), 1100 °C (**e**), 1200 °C (**f**), respectively. The scale bars in the SEM images indicate 500 nm. The insert yellow curves are the size distribution of LSM nanoparticles at the corresponding temperatures.

We further characterized the microstructure of LSM sintered bodies via SEM investigation. As shown in Figure 3, the sintering temperature significantly influences the microstructure of the LSM sintered body. The SEM image of the LSM dehydrated body in Figure 3a demonstrates that the raw LSM nanopowders with an average size of ~210 nm have been tightly fixed by the binder of agar (4% agar concentration). When sintering at 800 °C, the binder of agar was completely removed and consequently LSM powders congregated with a slight size growth from ~210 to ~225 nm, as shown in Figure 3b. When sintering at 900 °C, LSM powders began coalescing and the average size grew up to ~260 nm in Figure 3c. As the sintering temperature increased to 1000 °C, LSM powders coalesced to form porous structures with the average size exceeding 320 nm, as in Figure 3d. Figure 3e shows the microstructure of the LSM sintered body with the sintering temperature of 1100 °C. Obviously, the LSM powders tremendously grew into large blocks and their crystallinity significantly enhanced, whereas the porous structure disappeared in contrast with the LSM sintered body with the sintering temperature of 1000 °C. When the sintering temperature ascended to 1200 °C, LSM powders were sintered into a solid LSM ceramic monolith, as illustrated in Figure 3f. This indicates that high-temperature sintering treatment facilitates the densification of LSM ceramics.

### 3.3. Properties of LSM Sintered Bodies

**Figure 4:**
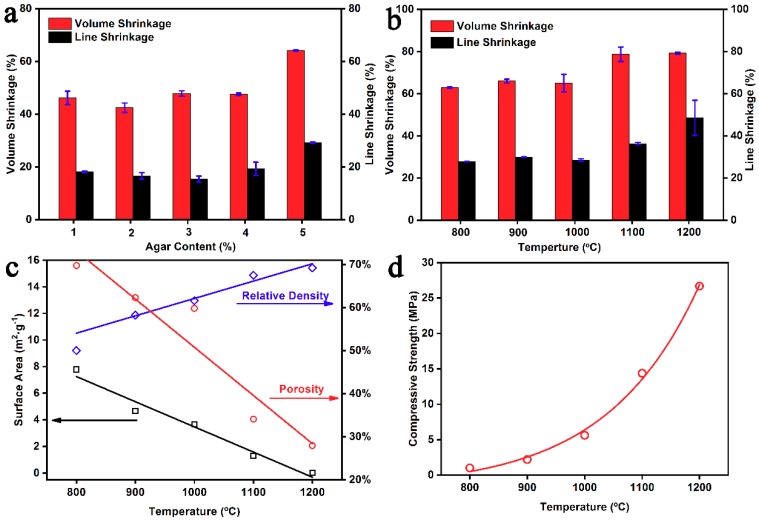
(**a**) The effect of agar concentration on the linear and volume shrinkage during dehydration; (**b**) the effect of sintering temperature on the linear and volume shrinkage during high-temperature sintering treatment; (**c**) the effect of sintering temperature on the specific surface area, porosity, and relative density of LSM sintered bodies; (**d**) the effect of sintering temperature on the compressive strength of LSM sintered bodies (the compression speed was 2 mm/min).

During the process of dehydration and high-temperature sintering treatment, we observed that the significant linear and volume shrinkage were closely related to the fabrication conditions, e.g., the agar content and the sintering temperature. Figure 4a shows the effects of the agar content during dehydration. When the agar content increased from 1% to 4%, the linear and volume shrinkage remained around 18% and 46%, respectively. However, the linear and volume shrinkage increased to ~29% and ~64%, respectively, when the agar content was 5%. This indicates that a small amount of agar content can make a great difference in the linear and volume shrinkage during dehydration. Moreover, the LSM green bodies with the 4% agar concentration were utilized to investigate the shrinkage during high-temperature sintering treatment. Figure 4b shows the effects of temperature during sintering treatment. As the sintering temperature rose from 800 °C to 1200 °C, the linear shrinkage gradually increased from ~27% to ~48% and the volume shrinkage gradually increased from ~62% to ~79%. This shows that high temperatures promote the linear and volume shrinkage during sintering treatment.

Furthermore, we measured the porosity, specific surface area and relative density of LSM sintered bodies at different sintering temperatures. First, the pore size distribution of LSM sintered bodies, as shown in Figure S2 (Supplementary Materials) were measured via mercury injection apparatus and the detailed average pore diameter were listed in Table S1 (Supplementary Materials). The average pore diameter gradually increased from 200.6 nm to 305.5 nm when the sintering temperature increased from 800 °C to 1000 °C, and dramatically increased to 604.4 nm and 1.72 μm when the sintering temperature was 1100 °C and 1200 °C, respectively. However, we note that the pore volume gradually decreased from ~0.6 mL/g to ~0.4 mL/g when the pore diameter was less than 500 nm, and then dramatically decreased to ~0.05 mL/g and ~0.004 mL/g when the sintering temperature was 1100 °C and 1200 °C, respectively. This strongly indicates that increasing temperatures benefit LSM nanopowders sintering. Obviously, the ultrasmall pore volume with the pore diameter less than 500 nm indicates the dense sintered LSM bodies, which is consistent with the SEM image of LSM body with the sintering temperature at 1200 °C. On the other hand, the pore size larger than 500 nm should be attributed to air holes and cracks which origin from the process of gel casting and high temperature sintering, respectively. Then, we calculated the porosity of LSM sintered bodies at different sintering temperatures. As shown in Figure 4c, the porosity of LSM sintered bodies is ~69% when the sintering temperature is 800 °C, and then it decreases to ~62% and ~60% when increasing the temperature to 900 °C and 1000 °C, respectively. Furthermore, the porosity dramatically decreased to ~34% and ~28% when the sintering temperature increased to 1100 °C and 1200 °C, respectively. This trend is consistent with the pore volume results. Moreover, we investigated the specific surface area of LSM sintered bodies via the BET method. In Figure 4c, the specific surface area decreased from 7.7 m^2^·g^−1^ to 0.000014 m^2^·g^−1^ when the sintering temperature increased from 800 °C to 1200 °C. Such declining trend is consistent with the result of porosity and the SEM investigation in Figure 3. Additionally, we measured the relative density of LSM sintered bodies at different sintering temperatures. Figure 4c demonstrates that the relative density gradually increased from ~50% to ~69% with the sintering temperature rising from 800 °C to 1200 °C, in accordance with the increase of linear and volume shrinkage in Figure 4b. We further tested the compressive strength of LSM sintered bodies at different sintering temperatures. As the sintering temperature rose from 800 °C to 1000 °C, the compressive strength of LSM sintered bodies increased exponentially from 2 MPa to 5.6 MPa. Then, the value rose sharply to 15 MPa at 1100 °C, and it increased to 25 MPa at 1200 °C. This trend may result from their increasingly solid structures [26].

## 4. Discussion

In a traditional acrylamide gel casting process, an irreversible free radical triggered polymerization was used to form gel bodies, which significantly depended on many reaction conditions such as concentration, temperature, pH values, etc. In comparison, a reversible agar gelation was utilized to form gel bodies during a typical agar gel casting process, which can be simply controlled by temperature tuning. When the temperature is lower than glass state temperature, agar aqueous solutions were solidified to agar gels, conversely, agar gels were melted to solutions when the temperature is higher than glass state temperature. This indicates that controlling the process of agar gelation is easier than that of acrylamide polymerization. Furthermore, APS is a sensitive radical generator that was used for acrylamide polymerization in the gel casting process. However, it could be decomposed by many transition metal oxides and thus limited the use of acrylamide polymerization for gel casting transition metal oxides. In contrast, agar is completely inert to transition metal oxides under mild conditions, which might be more suitable for gel casting transition metal contained ceramics. Moreover, acrylamide polymerization is an irreversible chemical reaction, whereas agar gelation is a reversible process that facilitates remolding gel bodies in the recycling process. In addition, agar-based gel bodies are able to manufacture in the secondary process, including cutting and drilling in meso-scale, which exhibits great potential for the fabrication of cathode-supported micro-SOFC.

High-temperature sintering treatment is another important process that directly determines the linear and volume shrinkage, specific surface area, porosity, relative density, and compressive strength of LSM ceramics. As the rise of sintering temperature, the specific surface area and porosity were descending, whereas the relative density and compressive strength were ascending. Coupling with the SEM images of LSM ceramics sintering at different temperatures, we propose a probable sintering mechanism to understand the high-temperature sintering treatment in Figure S3 (Supplementary Materials). At the initial stage of dehydrated body, LSM nanoparticles were tightly fixed in the agar gel skeleton at room temperature. When sintering at 800 °C, the agar skeleton was completely decomposed and the rest of LSM nanoparticles conglomerated and remained the shape of dehydrated body. Such LSM nanoparticles started to agglomeration when the sintering temperature reached 900 °C. A distinctive porous structure emerged with inter-connected pores and obvious agglomeration when the sintering temperature further increased to 1000 °C. When sintering at 1100 °C, the size of LSM particles dramatically increased and the crystallinity significantly enhanced as well. On the other hand, the porosity declined significantly, especially the pore size distribution in range from 200 to 500 nm. When the sintering temperature rose to 1200 °C, the porosity decreased continuously and the pores with the size distribution in range of 200~500 nm were eliminated, resulting in the steadily enhanced compressive strength which was able to meet the requirement of the Micro-SOFC electrode [27].

## 5. Conclusions

In summary, we have developed a low-cost and environmentally friendly LSM ceramics method for fabricating LSM ceramics with certain shapes via using agar gel casting and high temperature sintering. During the agar gel casting process, a physical method of temperature tuning was used to form gel bodies, rather than a chemical method of radical-triggered polymerization in a traditional acrylamide gel casting process. Based on such reversible temperature-tuning physical process, the as-fabricated LSM gel bodies facilitate not only manufacturing in the secondary process but also remolding and recycling during the gel casting process. During high-temperature sintering process, the relationships between the sintering temperature, microstructure, and the properties including the specific surface area, relative density, porosity, and compressive strength were discussed. Our studies are of potential meaning for the design and construction of high-temperature energy conversion and storage devices.

## Supplementary Materials

The following are available online at https://www.mdpi.com/1996-1944/12/6/848/s1, Figure S1: The TG curve of the LSM dehydrated body; Figure S2: Pores size distribution of LSM sintered bodies with different sintering temperatures; Figure S3: A diagram of LSM sintered bodies with different sintering temperatures; Table S1: The average pore diameter of sintered at different temperature.

## References

[1] Correa-Baena J.-P., Saliba M., Buonassisi T., Graetzel M., Abate A., Tress W., Hagfeldt A. (2017). Promises and challenges of perovskite solar cells. Science.

[2] Royer S., Duprez D., Can F., Courtois X., Batiot-Dupeyrat C., Laassiri S., Alamdari H. (2014). Perovskites as substitutes of noble metals for heterogeneous catalysis: dream or reality. Chem. Rev..

[3] Onrubia J.A., Pereda-Ayo B., De-La-Torre U., González-Velasco J.R. (2017). Key factors in Sr-doped LaBO_3_ (B = Co or Mn) perovskites for NO oxidation in efficient diesel exhaust purification. Appl. Cataly. B..

[4] Zhao Y.J., Hang Y., Zhang Y.D., Wang Z.D., Yao Y.J., He X.J., Zhang C.F., Zhang D.W. (2017). Strontium-doped perovskite oxide La_1−x_Sr_x_MnO_3_ (x = 0, 0.2, 0.6) as a highly efficient electrocatalyst for nonaqueous LiO_2_ batteries. Electrochimica Acta.

[5] Song S., Zhou J., Zhang S., Zhang L., Li J., Wang Y., Han L., Long Y., Hu Z., Wang J.-Q. (2018). Molten-salt synthesis of porous La_0.6_Sr_0.4_Co_0.2_Fe_0.8_O_2.9_ perovskite as an efficient electrocatalyst for oxygen evolution. Nano Res..

[6] Zhi M., Zhou G., Hong Z., Wang J., Gemmen R., Gerdes K., Manivannan A., Ma D., Wu N. (2011). Single crystalline La_0.5_Sr_0.5_MnO_3_ microcubes as cathode of solid oxide fuel cell. Energy Environ. Sci..

[7] Javanshir E., Mozammel M., Tanhaei M. (2018). Fabrication of improved La_0.8_Sr_0.2_MnO_3_ cathode layer for solid oxide fuel cells using economical coating methods. Int. J. Appl. Ceram. Technol..

[8] Hedayat N., Du Y., Ilkhani H. (2017). Review on fabrication techniques for porous electrodes of solid oxide fuel cells by sacrificial template methods. Renew. Sustain. Energy Rev..

[9] Haleem B.A., Bhuvana R., Udayakumar A. (2009). Gelcasting of Strontium Doped Lanthanum Manganite for Solid Oxide Fuel Cell Applications. Trans. Indian Ceram. Soc..

[10] Xiao W., Wang D. (2014). The electrochemical reduction processes of solid compounds in high temperature molten salts. Chem. Soc. Rev..

[11] Licht S., Cui B., Stuart J., Wang B., Lau J. (2013). Molten air—A new, highest energy class of rechargeable batteries. Energ. Environ. Sci..

[12] Peng C., Guan C., Lin J., Zhang S., Bao H., Wang Y., Xiao G., Chen G.Z., Wang J.-Q. (2018). A Rechargeable High-Temperature Molten Salt Iron-Oxygen Battery. ChemSusChem.

[13] Stoever D., Buchkremer H.P., Mai A., Menzler N.H., Zahid M. (2007). Processing and properties of advanced solid oxide fuel cells. Mater. Sci. Forum.

[14] Peng Z.Y., Liu M.L. (2001). Preparation of dense platinum-yttria stabilized zirconia and yttria stabilized zirconia films on porous La_0.9_Sr_0.1_MnO_3_ (LSM) substrates. J. Am. Ceram. Soc..

[15] Chen G., You H.-X., Kasai Y., Sato H., Abudula A. (2011). Characterization of planer cathode-supported SOFC prepared by a dual dry pressing method. J. Alloy. Compd..

[16] Piao J., Sun K., Zhang N., Xu S. (2008). A study of process parameters of LSM and LSM-YSZ composite cathode films prepared by screen-printing. J. Power. Sources.

[17] Yuan C., Liu Y., Zhou Y., Zhan Z., Wang S. (2013). Fabrication and characterization of a cathode-support solid oxide fuel cell by tape casting and lamination. Int. J. Hydrog. Energy.

[18] Deng T., Wang Y., Dufresne A., Lin N. (2018). Simultaneous enhancement of elasticity and strength of Al_2_O_3_-based ceramics body from cellulose nanocrystals via gel-casting process. Carbohyd. Polym..

[19] Munro C.D., Plucknett K.P. (2011). Agar-Based Aqueous Gel Casting of Barium Titanate Ceramics. Int. J. Appl. Ceram. Tecnol..

[20] Tulliani J.-M., Bemporad E., Sebastiani M., Pulci G., Tirillo J., Bartuli C. (2013). Dense and Cellular Zirconia Produced by Gel Casting with Agar: Preparation and High Temperature Characterization. J. Nanomater..

[21] Yamaguchi T., Shimizu S., Suzuki T., Fujishiro Y., Awanoz M. (2009). Design and Fabrication of a Novel Electrode-Supported Honeycomb SOFC. J. Am. Ceram. Soc..

[22] Sun Y., Qin X., Zhou G., Zhang H., Peng X., Wang S. (2015). Gelcasting and reactive sintering of sheet-like YAG transparent ceramics. J. Alloy. Compd..

[23] Gilissen R., Erauw J.P., Smolders A., Vanswijgenhoven E., Luyten J. (2000). Gelcasting, a near net shape technique. Mater. Desig..

[24] Hammel E.C., Campa J.A., Armbrister C.E., Scheiner M.V., Okoli O.I. (2017). Influence of osmotic drying with an aqueous poly(ethylene glycol) liquid desiccant on alumina objects gelcast with gelatin. Ceram. Int..

[25] Cai Q., Teng F., Ning X., Zhang X. (2008). Preparation of Macroporous Ceramics Using Agar Gelcasting Foam Method. Rare Metal Mater. Eng..

[26] Rugele K., Lehmhus D., Hussainova I., Peculevica J., Lisnanskis M., Shishkin A. (2017). Effect of Fly-Ash Cenospheres on Properties of Clay-Ceramic Syntactic Foams. Materials.

[27] Fang X., Zhu J., Lin Z. (2018). Effects of Electrode Composition and Thickness on the Mechanical Performance of a Solid Oxide Fuel Cell. Energies.

