# Supplementary material for: Fabrication of Lanthanum Strontium Manganite Ceramics via Agar Gel Casting and Solid State Sintering
Source: Materials (Basel). 2019 Mar 13;12(6):848. doi: 10.3390/ma12060848 (PMC6472146; doi:10.3390/ma12060848)
Supplement: Supplementary file 1 [file materials-12-00848-s001.pdf]

Supplementary Materials: Fabrication of Lanthanum Strontium Manganite Ceramics via Agar Gel Casting and Solid State Sintering

Shiyu Zhang [1,2], Cheng Peng [1,*], Chengzhi Guan [1], Guoping Xiao [1] and Jianqiang Wang [1,2,*]

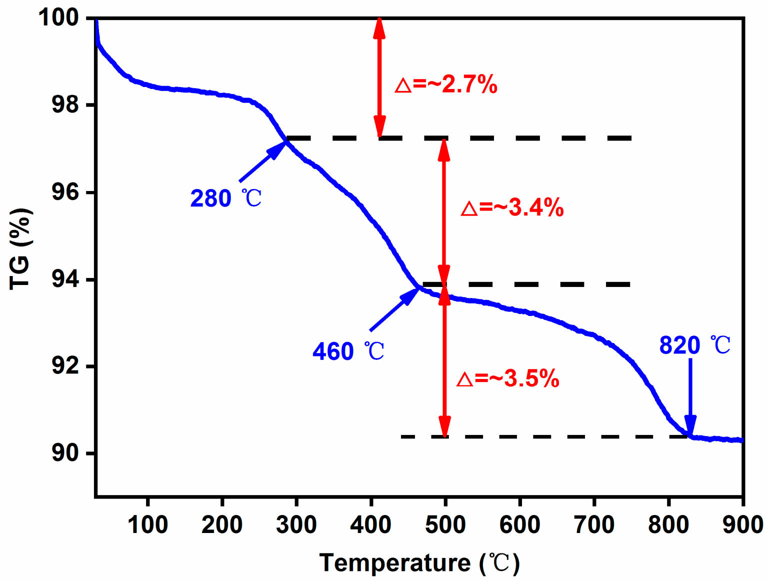

Figure S1. The TG curve of the LSM dehydrated body.

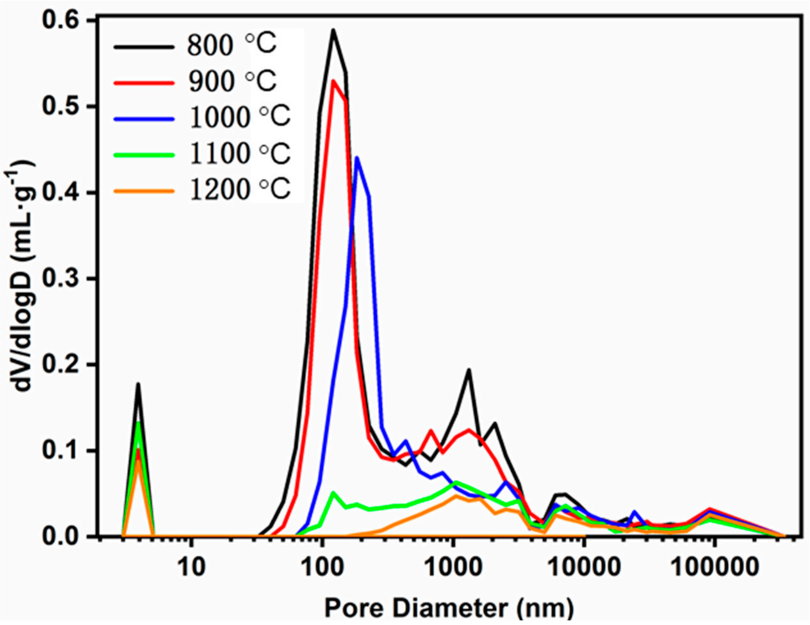

Figure S2. Pores size distribution of LSM sintered bodies with different sintering temperatures.

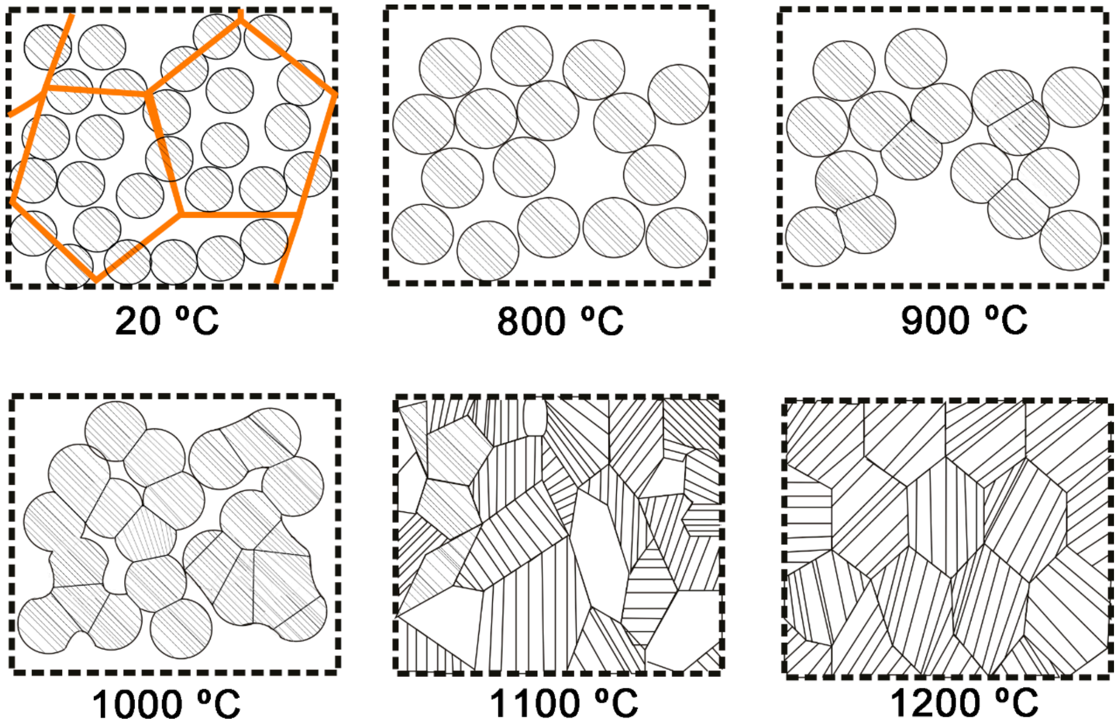

Figure S3. A diagram of LSM sintered bodies with different sintering temperatures.

Table S1. The average pore diameter of sintered at different temperature

**Temperature**	**800 °C**	**900 °C**	**1000 °C**	**1100 °C**	**1200 °C**
Average pore diameter (nm)	200.6	223.7	305.5	604.4	1723.3
